# Case Report: Assessing the Position of Pacemaker Leads *via* Transthoracic Echocardiography: Additional Value of the Subcostal En Face View

**DOI:** 10.3389/fcvm.2021.697052

**Published:** 2021-06-04

**Authors:** Andrea Simone Deichl, Philipp Lacour, Evgeny Belyavskiy, Burkert Pieske, Elisabeth Pieske-Kraigher, Florian Blaschke, Matthias Schneider

**Affiliations:** ^1^Department of Internal Medicine and Cardiology, Charité – Universitätsmedizin Berlin (Campus Virchow-Klinikum), Berlin, Germany; ^2^DZHK (German Center for Cardiovascular Research) Partner Site Berlin, Berlin, Germany; ^3^Berlin Institute of Health (BIH), Berlin, Germany; ^4^German Heart Center Berlin, Berlin, Germany

**Keywords:** pacemaker, echocardiography, tricuspid regurgitation, en-face imaging, tricuspid valve

## Abstract

There is an association between presence of cardiac implantable electronic devices (CIED) and development of tricuspid regurgitation (TR). Mechanisms proposed to explain CIED-induced TR can be classified as implantation-related, lead-related, and pacing-related. Lead-related TR results from the direct interaction of the lead with the tricuspid valve (TV). The localization of the lead at the TV level directly influences the probability of subsequent development of significant TR. A transthoracic subcostal en face view of the TV can be acquired in most patients through a 90° rotation from the subcostal 4-chamber view with clear anatomic delineation of the TV and the commissures including lead position. This case-series presents three examples where the transthoracic en face view could add incremental information on the position of the pacemaker leads and on the mechanism of TR.

**Conclusion:** When performing transthoracic echocardiography in patients with trans-tricuspid CIED lead(s), an en face view of the TV with exact reporting of the position of the lead(s) should be included.

## Pacemaker Lead Associated Tricuspid Regurgitation

The first permanent electronic pacemaker (PM) was implanted by Senning and Elmqvist in Sweden in 1958 using a thoracotomy to suture two epicardial electrodes (1). Since that day, therapy with cardiac implantable electronic devices (CIED) has impressively advanced, notably with the advent of automatic cardioverter defibrillators (ICD) to prevent sudden arrhythmic death, cardiac resynchronization therapy (CRT), and cardiac contractility modulation (CCM) as therapy strategies for chronic heart failure. Due to an aging population and the increasing use of CIED in this patient group, possible complications need to be focused on as well. The reported frequency of developing significant TR following CIED implantation varies, ranging from 7 to 45% (2). An increase of TR after placing of an RV lead is associated with a dismal outcome (3). Mechanisms proposed to explain CIED-induced TR can be classified as implantation-related, lead-related, and pacing-related (2). Another classification proposes a distinction between primary (implantation-related, lead-related) and secondary (pacing related, “functional”) CIED-induced TR. Primary CIED-induced TR results from the direct interaction of the lead with the tricuspid valve. The underlying mechanism for secondary CIED-induced TR is characterized by right ventricular (RV) dilatation and dysfunction e.g., caused by a high percentage of RV pacing (4, 5).

The localization of the lead at the tricuspid valve level directly influences the probability of subsequent development of significant TR. Particularly antero-septal and antero-posterior commissural positions cause TR, while postero-septal commissural or central lead positions are less often associated with the development of TR (2).

## Echocardiographic Assessment of Patients With CIED

Echocardiographic assessment of a patient with CIED can be performed by transthoracic (TTE) and transesophageal echocardiography (TOE). The following points need to be addressed:

- Morphology of the tricuspid valve (tricuspid? bicuspid? indentations?)
- Presence/severity of TR
- Number of CIED leads
- Where do(es) the RV lead(s) pass the tricuspid valve (commissural? If yes: which commissure? Central? Is there leaflet impingement or perforation?).

In most cases, TTE is sufficient to comprehensively describe both degree of TR and position of the lead(s). In rare cases, additional TOE is necessary to complete the assessment.

## TTE

In ultrasound in general but in the assessment of the tricuspid valve in particular, a combination of different views needs to be combined. These include the parasternal long and short axis RV inflow views, the apical 4-chamber view with anterior and posterior angulation, the apical long- axis RV inflow view, and the subcostal 4-chamber view (6–9). In the parasternal short axis, a commissural view of the TV can be acquired with the antero-septal commissure on the right (in proximity to the aortic valve), and the postero-septal commissure on the left side of the screen.

In none of the mentioned angulations, the entire TV with all its leaflets can be evaluated simultaneously. Therefore, particularly if more than one RV lead is present, the assessment can be inconclusive.

**Figure 1:**
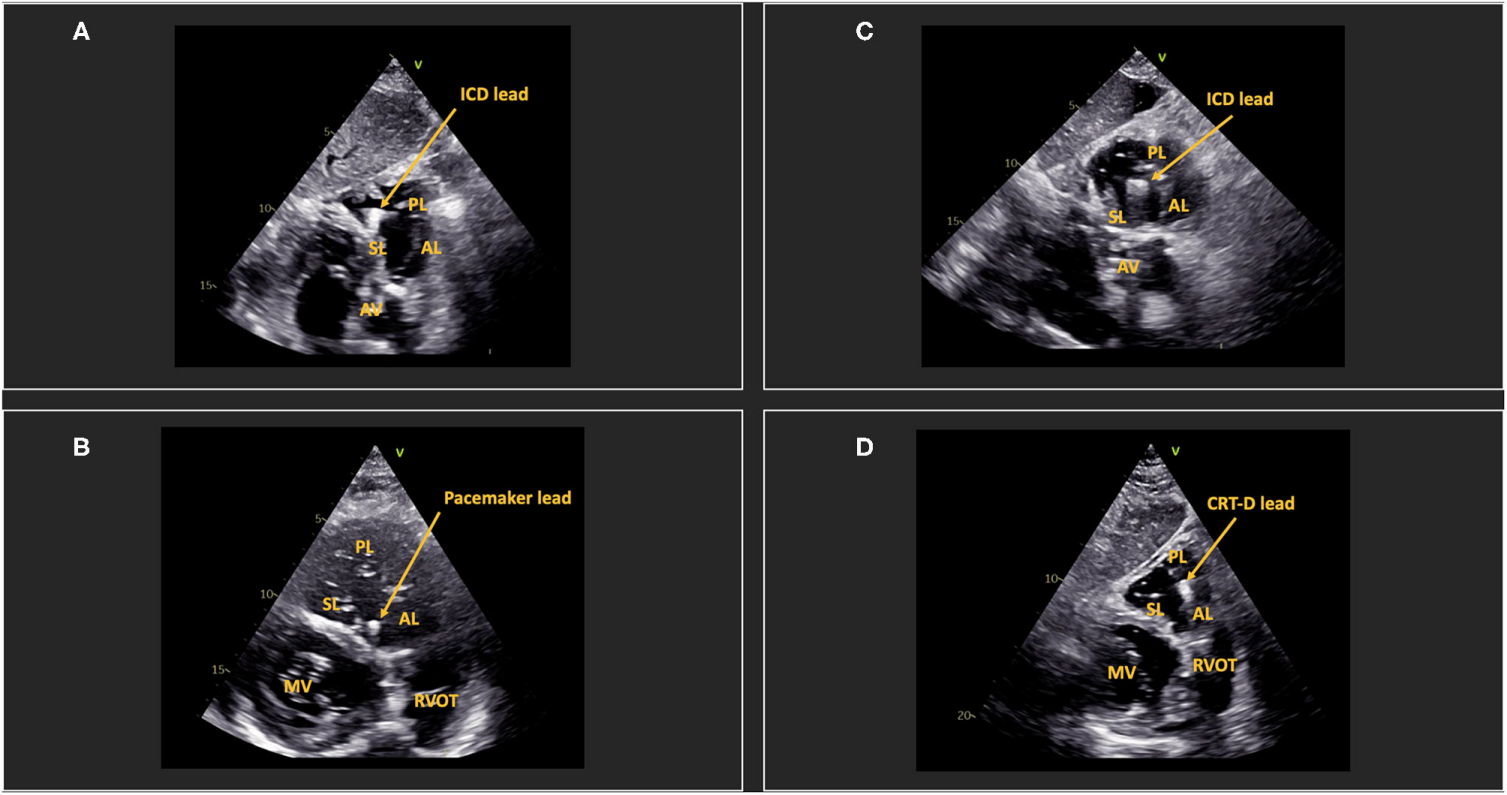
Determination of lead position *via* subcostal en face view of the tricuspid valve. **(A)** shows an ICD lead in the postero-septal commissure. **(B)** shows a pacemaker lead in the anteroseptal commissure. **(C)** shows an ICD lead in central position. **(D)** shows a CRT-D lead in the posterior/anterior commissure. AL, anterior leaflet; PL, posterior leaflet; SL, septal leaflet; MV, mitral valve; RVOT, right ventricular outflow tract; AV, aortic valve; ICD, implantable cardioverter-defibrillator; CRT-D, Cardiac resynchronization therapy-defibrillator.

An additional atypical subcostal en face view of the tricuspid valve can be acquired in most patients and has previously been shown to clarify CIED lead position and mechanism of TR in small studies and case series (10, 11). The view can be achieved by a 90° rotation from the regular subcostal 4-chamber view. A clear anatomic delineation of the TV and its commissures can be visualized (Figure 1). Pacemaker lead position can be described accurately in most patients (11). Important anatomical landmarks are the interventricular septum (septal leaflet), the aortic valve (at the antero-septal commissure), and the right ventricular outflow tract (at the anterior leaflet) (Figure 1). The coronary sinus is in proximity to the postero-septal commissure (12, 13).

Ideally, an additional 3D en face view of the TV should be included in the assessment, however this may be limited to patients with good apical image quality (14).

## TOE

In many cases, TOE is not necessary to completely understand the mechanisms of TR. However, TOE may provide superior image quality of the tricuspid valve. Two angulations are crucial: A deep-transesophageal 70° angulation provides a commissural view of the TV. A similar view can oftentimes be achieved in a transthoracic parasternal short axis as mentioned above. With biplane mode, the antero-septal and the postero-septal commissures can be visualized clearly. The second angulation is a 30° transgastric view which provides an en face view of the TV (15). This view is similar to the transthoracic subcostal en face view.

Considering the invasive nature and the need for sedation in most patients if transgastric angulations are attempted, this examination should only be performed in case of insufficient image quality in TTE.

## Examples of Lead-Induced Tricuspid Regurgitation

### Case 1: Septal Leaflet Impingement by Pacemaker Lead

**Figure 2:**
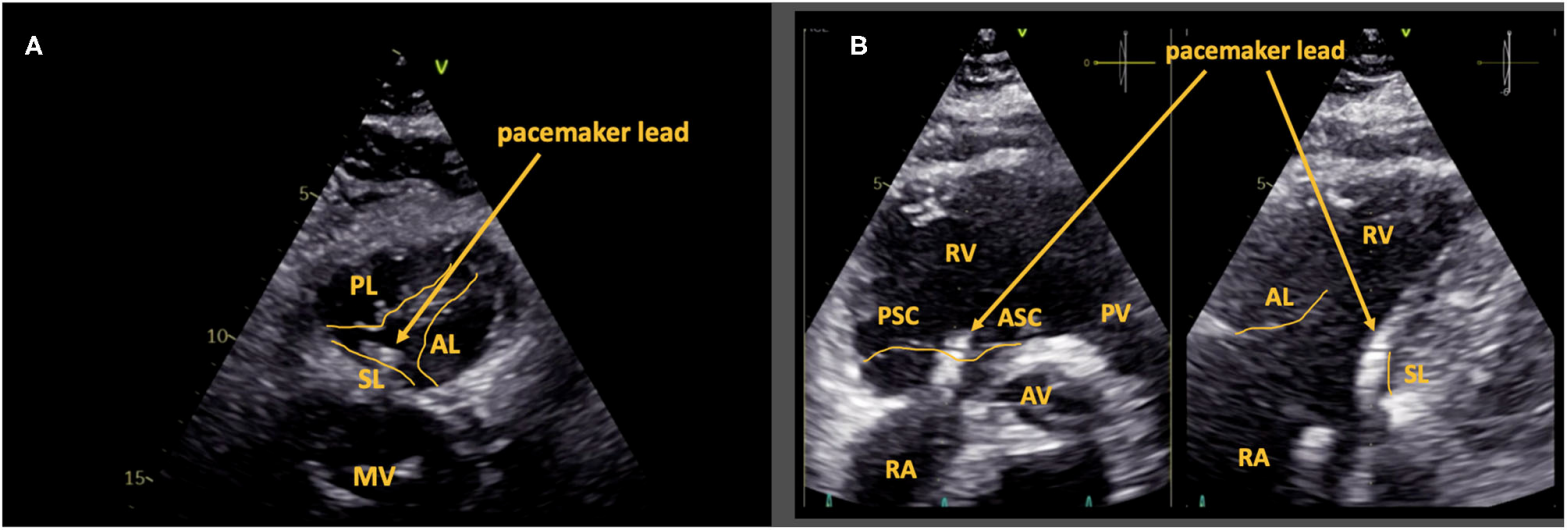
Case 1: Septal leaflet impingement by pacemaker lead. Central lead position with impingement of the septal leaflet is shown *via* subcostal en face view of the tricuspid valve **(A)** and *via* the commissural view in the parasternal short axis (**B**; left side) and the corresponding 90° view of the septal and the anterior leaflet (**B**; right side). AL, anterior leaflet; PL, posterior leaflet; SL, septal leaflet; RA, right atrium; RV, right ventricle; PSC, postero-septal commissure; ASC, antero-septal commissure; MV, mitral valve; PV, pulmonary valve; AV, aortic valve.

This 84 year old female patient received a 2-chamber pacemaker system due to complete heart block 12 years ago. The RV lead is located centrally at the septum, hindering the septal leaflet from coaptation. This results in severe TR. The exact lead position can be determined by TTE (Figure 2), both in an en face view (Figure 2A) and in a biplane angulation with a TV commissural view in the parasternal short axis (Figure 2B, left side) and the corresponding 90° view of the septal and the anterior leaflet (Figure 2B, right side). This patient presented with right heart decompensation due to severe structural tricuspid regurgitation (TR). She was subsequently evaluated for lead extraction.

### Case 2: Leaflet Perforation

**Figure 3:**
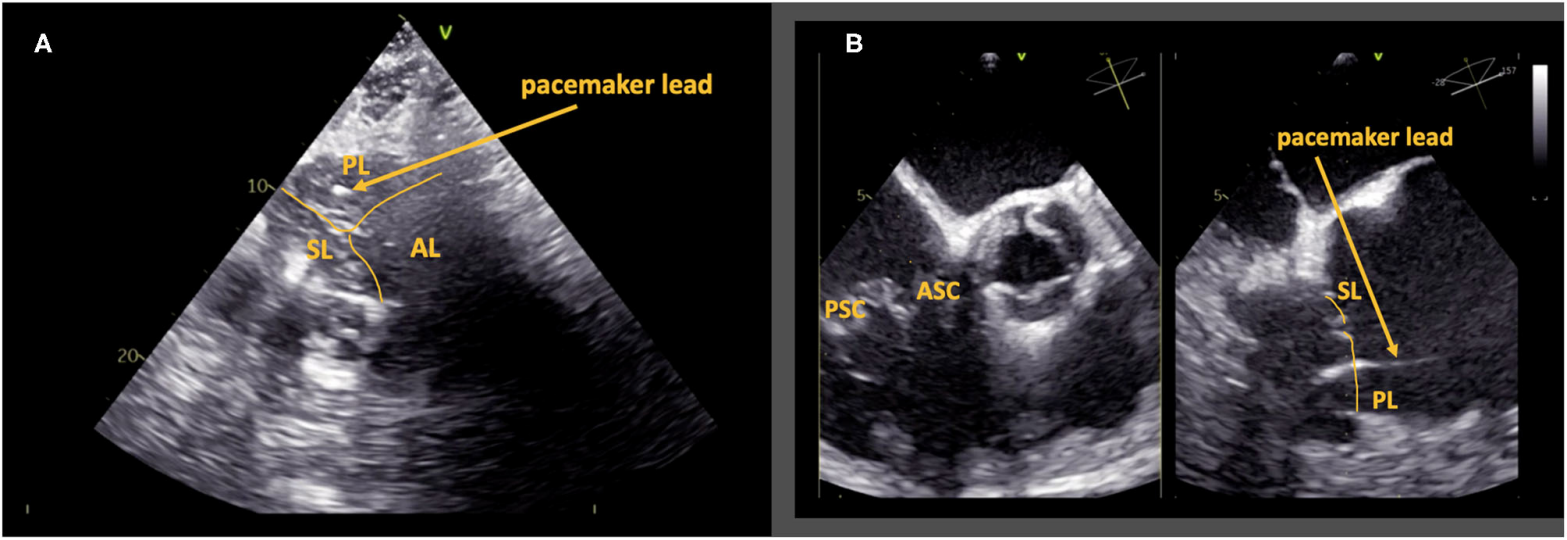
Case 2: Leaflet perforation. Transthoracic en face view of the tricuspid valve shows the pacemaker lead passing through the posterior leaflet **(A)**. In transesophageal echocardiography, a deep-transesophageal commissural view of the tricuspid valve (**B**, left side) with the corresponding 90° view of the septal and the posterior leaflet (**B**, right side) proves perforation of the posterior leaflet. AL, anterior leaflet; PL, posterior leaflet; SL, septal leaflet; PSC, postero-septal commissure; ASC, antero-septal commissure.

This 82 year old male patient received a 1-chamber pacemaker system due to atrial fibrillation with symptomatic bradycardia. A transthoracic en face view of the tricuspid valve showed the pacemaker lead passing through the posterior leaflet, which was highly suggestive of leaflet perforation. This could be proven by TOE (Figure 3). The patient did not have severe TR and did not report clinical signs of heart failure. Close follow-up examinations were scheduled.

### Case 3: Anterior Leaflet Impingement by a Pacemaker Lead

**Figure 4:**
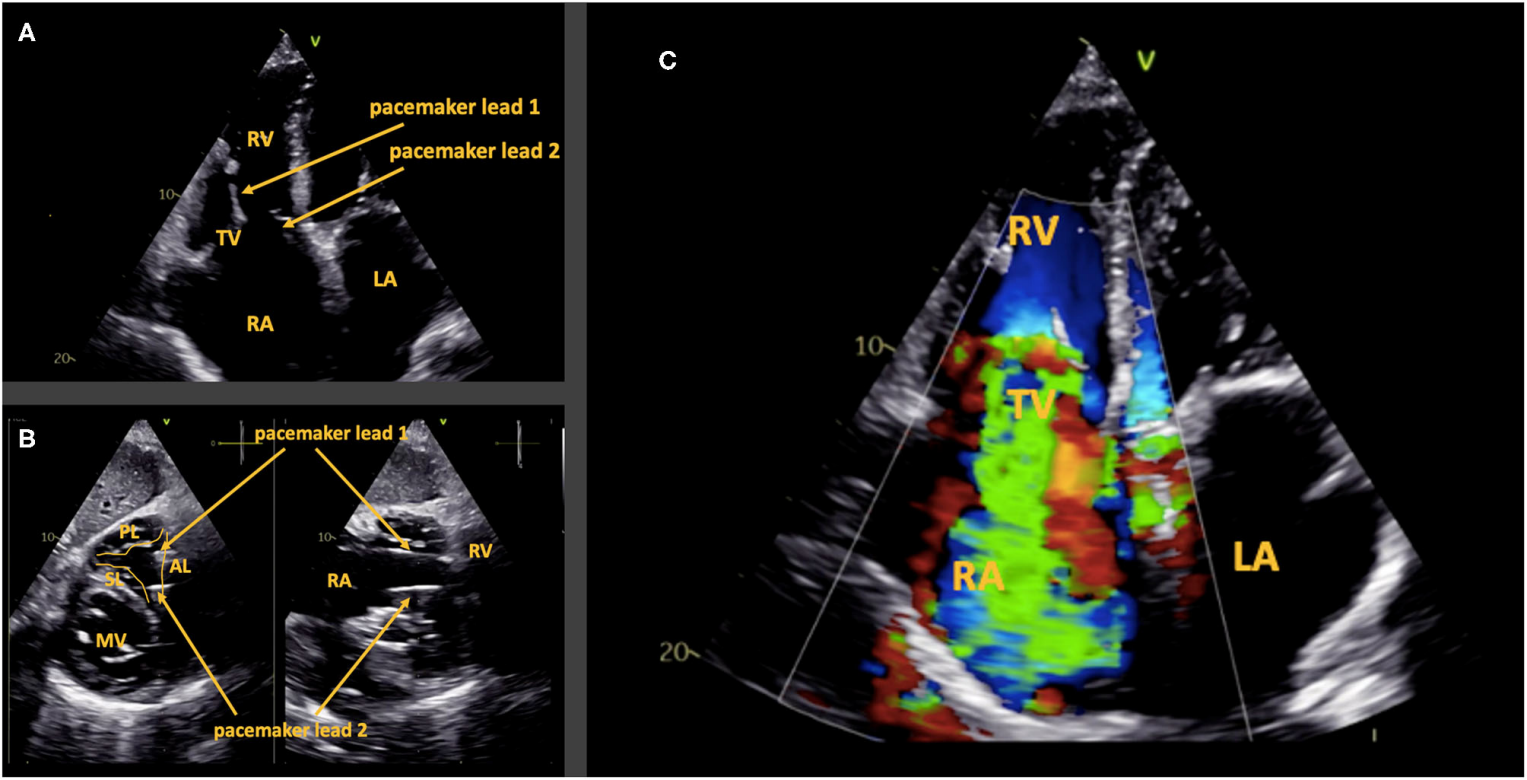
Case 3: Anterior leaflet impingement by a pacemaker lead. Two trans-tricuspid pacemaker leads are present **(A)**. The transthoracic subcostal en face view of the tricuspid valve shows one lead in the antero-posterior commissure and the other in the antero-septal commissure **(B)**. Leaflet impingement of the anterior leaflet causes “torrential” tricuspid regurgitation **(C)**. AL, anterior leaflet; PL, posterior leaflet; SL, septal leaflet; RA, right atrium; RV, right ventricle; LA, left atrium; TV, tricuspid valve; MV, mitral valve.

This 85 year old female patient received a second RV pacemaker lead due to dysfunction of the first lead. Transthoracic en face view shows the first pacemaker lead impinging the anterior leaflet causing a large coaptation defect with subsequent “torrential” TR. The second pacemaker lead is located in the antero-septal commissure (Figure 4). The patient presented with peripheral edema. She declined further evaluation and interventional treatment.

### Clinical Consequences

Severe tricuspid regurgitation is associated with dismal outcome (16). In recent years, interventional treatment of TR has emerged, allowing treatment of patients even if severe comorbidities are present (17). A small study indicates that patients with CIED related TR can also be treated successfully (18). However, it is crucial to determine if trans-tricuspid leads are secondary bystanders or primarily causing TR. The latter should be evaluated for lead extraction rather than interventional or surgical treatment. The proposed en-face view adds an important angulation to make the right diagnosis.

## Conclusion

When performing transthoracic echocardiography in patients with trans-tricuspid CIED lead(s), an en face view of the TV with exact reporting of the position of the lead(s) should be included. Future prospective studies should focus on the impact of lead position on subsequent development of TR and right heart remodeling.

## Data Availability Statement

The original contributions presented in the study are included in the article/supplementary material, further inquiries can be directed to the corresponding author.

## Author Contributions

AD, PL, FB, and MS contributed to conception and design of this analysis. AD and MS wrote the first draft of the manuscript. All authors contributed to manuscript revision, read, and approved the submitted version.

## Conflict of Interest

The authors declare that the research was conducted in the absence of any commercial or financial relationships that could be construed as a potential conflict of interest.
